# Supplementary material for: Proteomic and clinical biomarkers for acute mountain sickness in a longitudinal cohort
Source: Commun Biol. 2022 Jun 6;5:548. doi: 10.1038/s42003-022-03514-6 (PMC9170681; doi:10.1038/s42003-022-03514-6)
Supplement: Supplementary file 2 — Supplementary Figures [file 42003_2022_3514_MOESM2_ESM.pdf]

Proteomic and clinical biomarkers for acute mountain sickness in a longitudinal cohort

Jing Yang[1,2,3,4,+], Zhilong Jia[3,4,5,+,*], Xinyu Song[3,5], Jinlong Shi[2,3], Xiaoreng Wang[6], Xiaojing Zhao[4,7], Kunlun He[1,2,3,4,*]

[1] Medical School of Chinese PLA, Chinese PLA General Hospital, Beijing, China, 100853

[2] Research Center for Medical Big Data, Medical Innovation Research Division of Chinese PLA General Hospital, Beijing, China, 100853

[3] Key Laboratory of Biomedical Engineering and Translational Medicine, Ministry of Industry and Information Technology, Chinese PLA General Hospital, Beijing, China, 100853

[4] Beijing Key Laboratory of Chronic Heart Failure Precision Medicine, Chinese PLA General Hospital, Beijing, China, 100853

[5] Center for Artificial Intelligence in Medicine, Medical Innovation Research Division of Chinese PLA General Hospital, Beijing, China, 100853

[6] Laboratory of Radiation Injury Treatment, Medical Innovation Research Division, PLA General Hospital, Beijing, China, 100853

[7] Translational Medicine Research Center, Medical Innovation Research Division of Chinese PLA General Hospital, Beijing, China, 100853

[+] These authors contributed equally.

[*] Corresponding authors: Kunlun He (kunlunhe@plagh.org) and Zhilong Jia (jiazhilong@plagh.org)

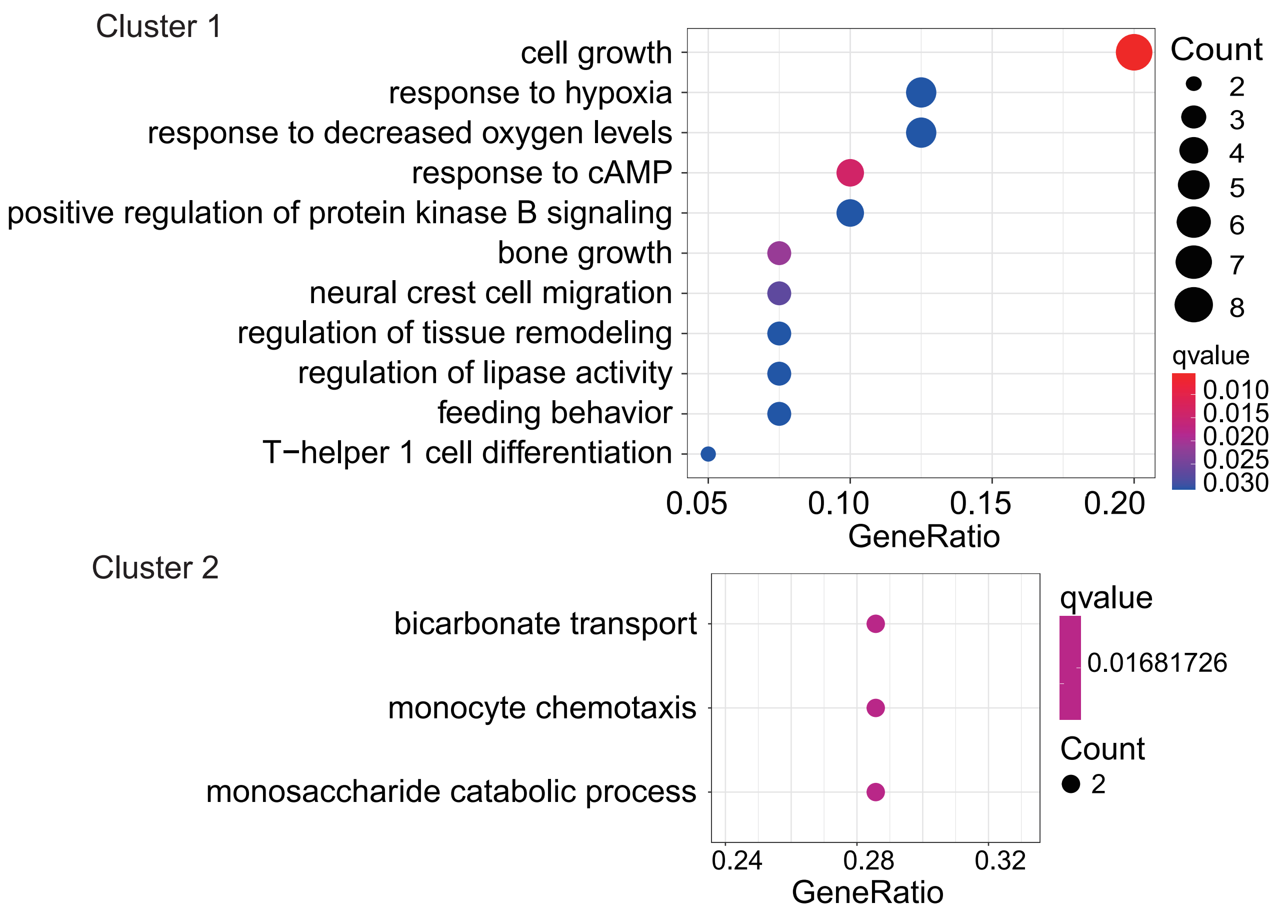

Supplementary Fig. 1

Dot plot of biological process-related Gene Ontology enrichment of the two clusters.
Terms with a q-value less than 0.05 are shown. Cell growth and response to stimulus were mainly enriched in cluster 1 (upper panel), and bicarbonate transport was mainly enriched in cluster 2 (lower panel).

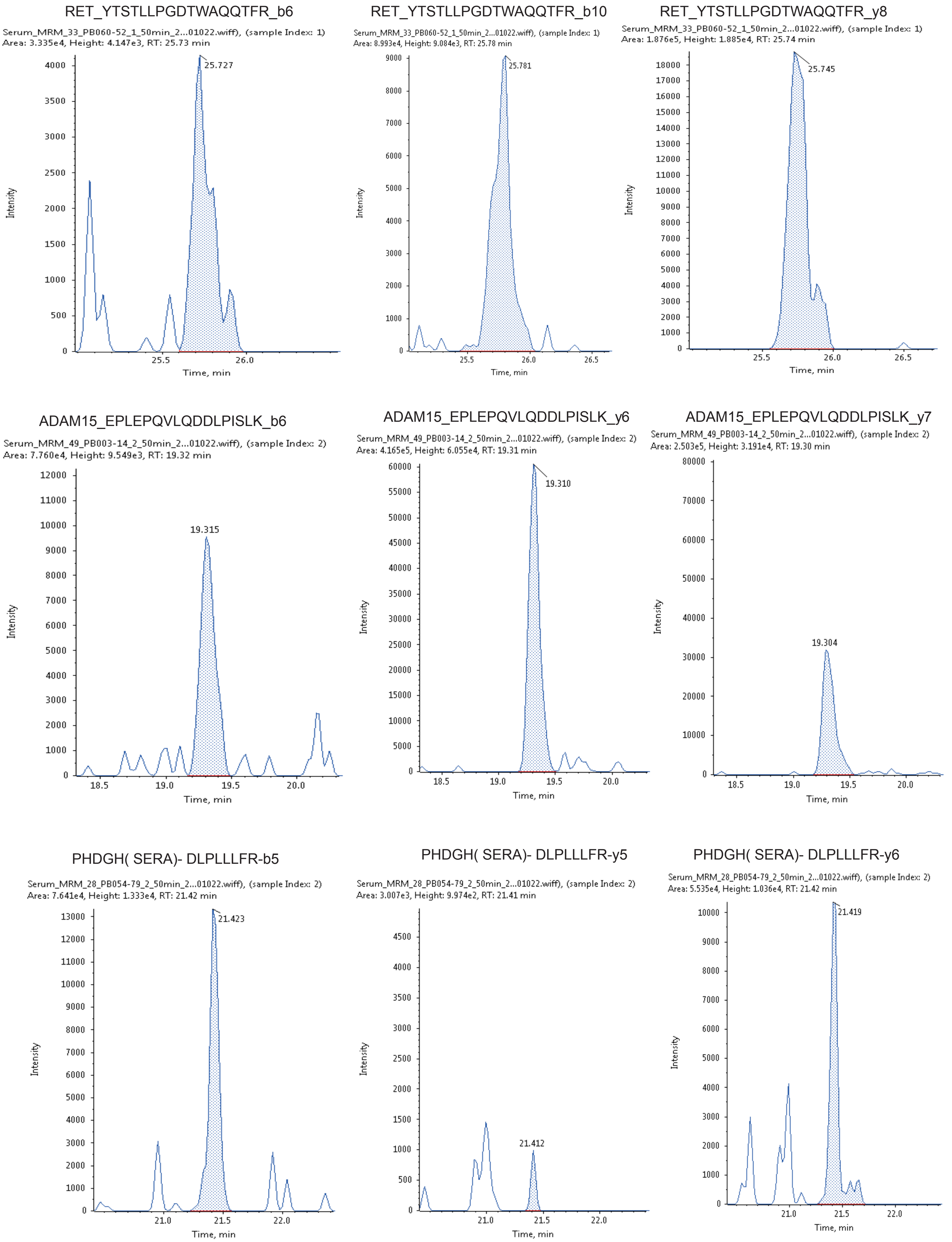

Supplementary Fig. 2

The sample extracted ion chromatograms of proteins measured by MRM.

The extracted ion chromatograms of RET, ADAM15, and PHGDH measured by MRM.

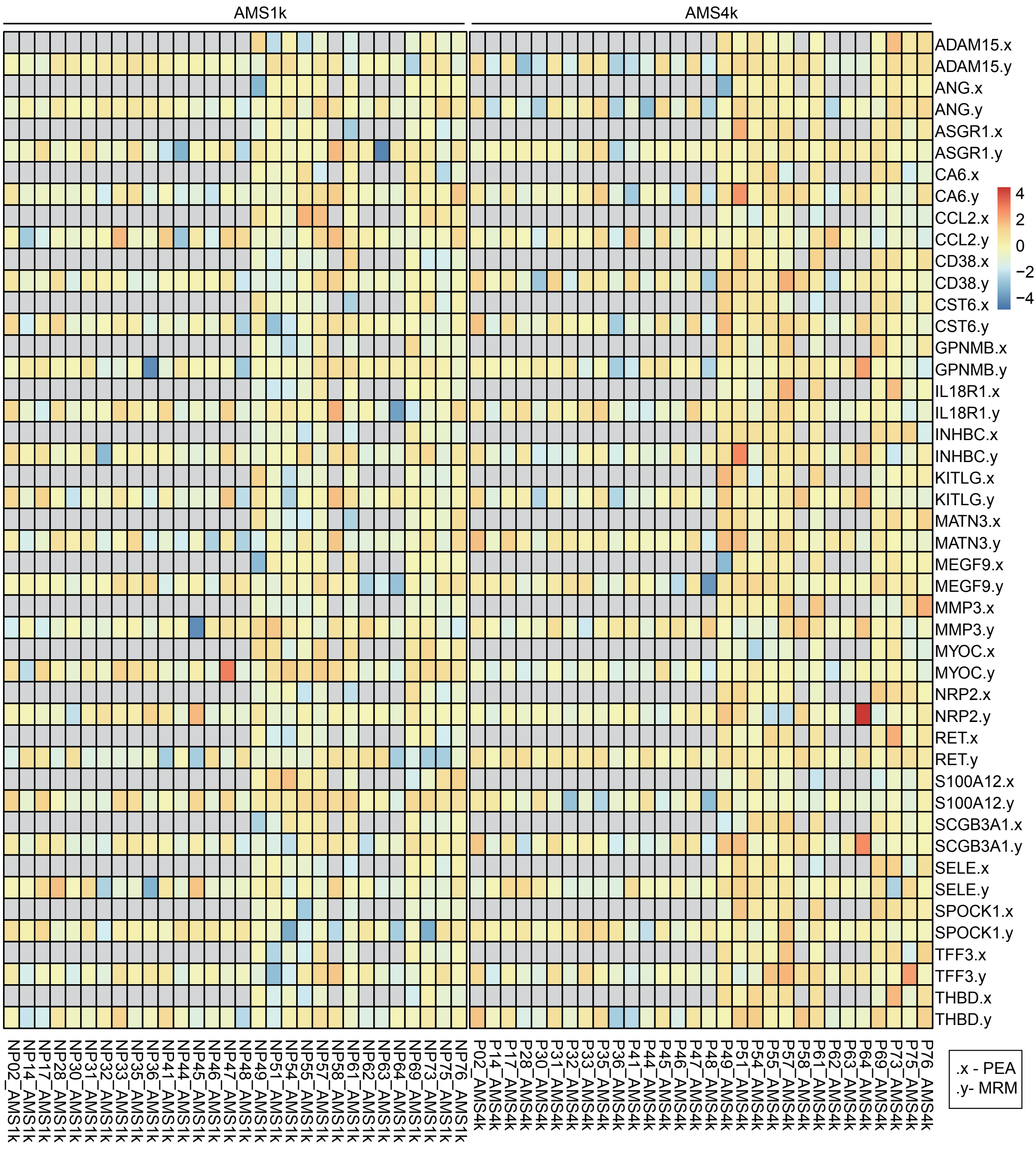

Supplementary Fig. 3

Abundance heatmap of 23 proteins measured by PEA and MRM in pathogenesis comparison. Proteins measured by PEA are shown with '.x', while by MRM are shown with '.y'. The gray boxes in the heatmap indicate that the samples were not measured by PEA.

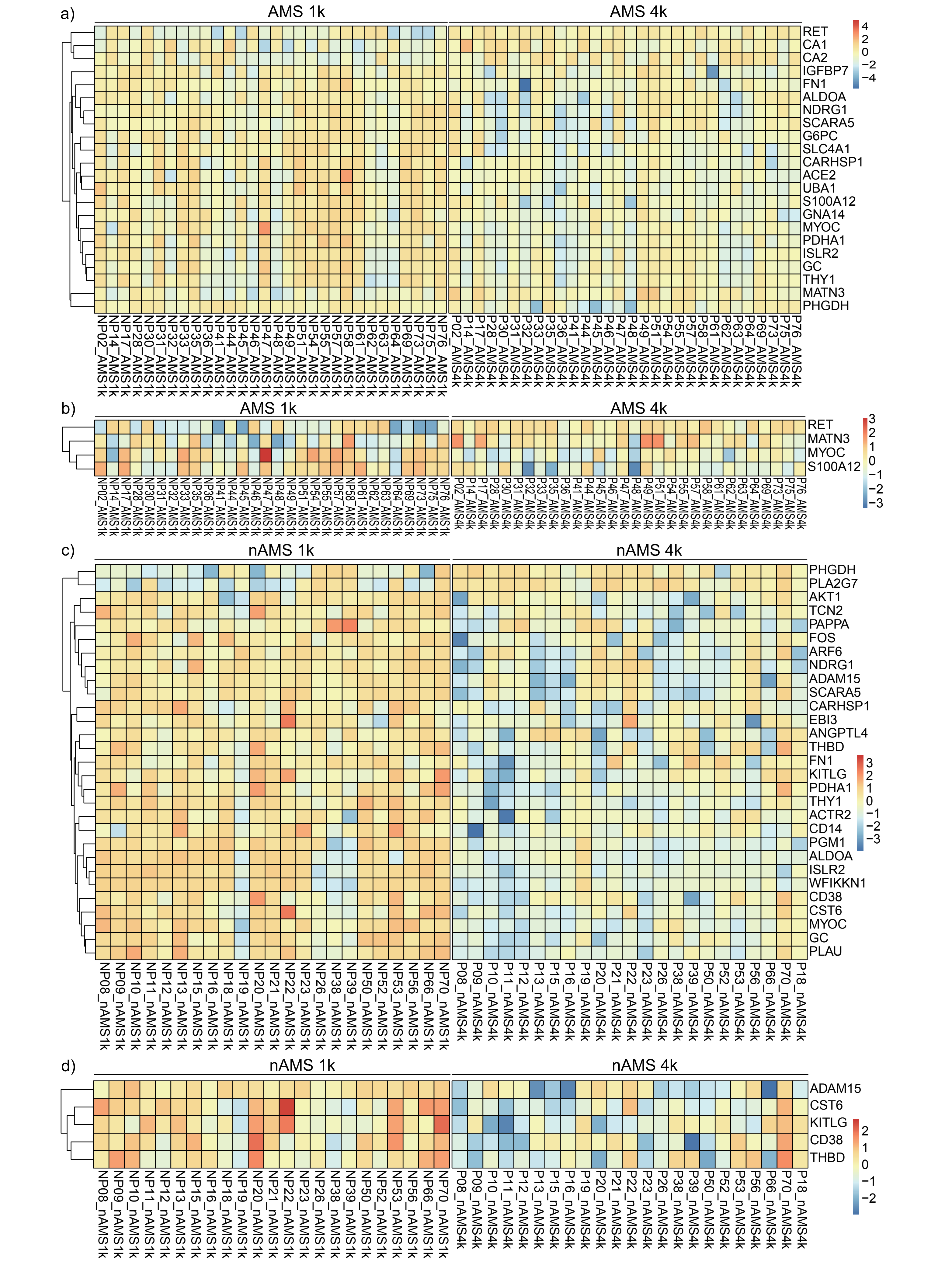

Supplementary Fig. 4 Heatmap of differentially expressed proteins in pathogenesis and protection comparison.
a) Heatmap of 22 differentially expressed proteins (q-values < 0.05) in pathogenesis comparison validated by MRM.
b) Heatmap of 4 differentially expressed proteins (q-values < 0.05) with the same regulation trends measured by both PEA and MRM in the pathogenesis comparison.
c) Heatmap of 29 differentially expressed proteins (q-values < 0.05) in protection comparison validated by MRM.
d) Heatmap of 5 differentially expressed proteins (q-values < 0.05) with the opposite regulation trends measured by PEA and MRM in the protection comparison.

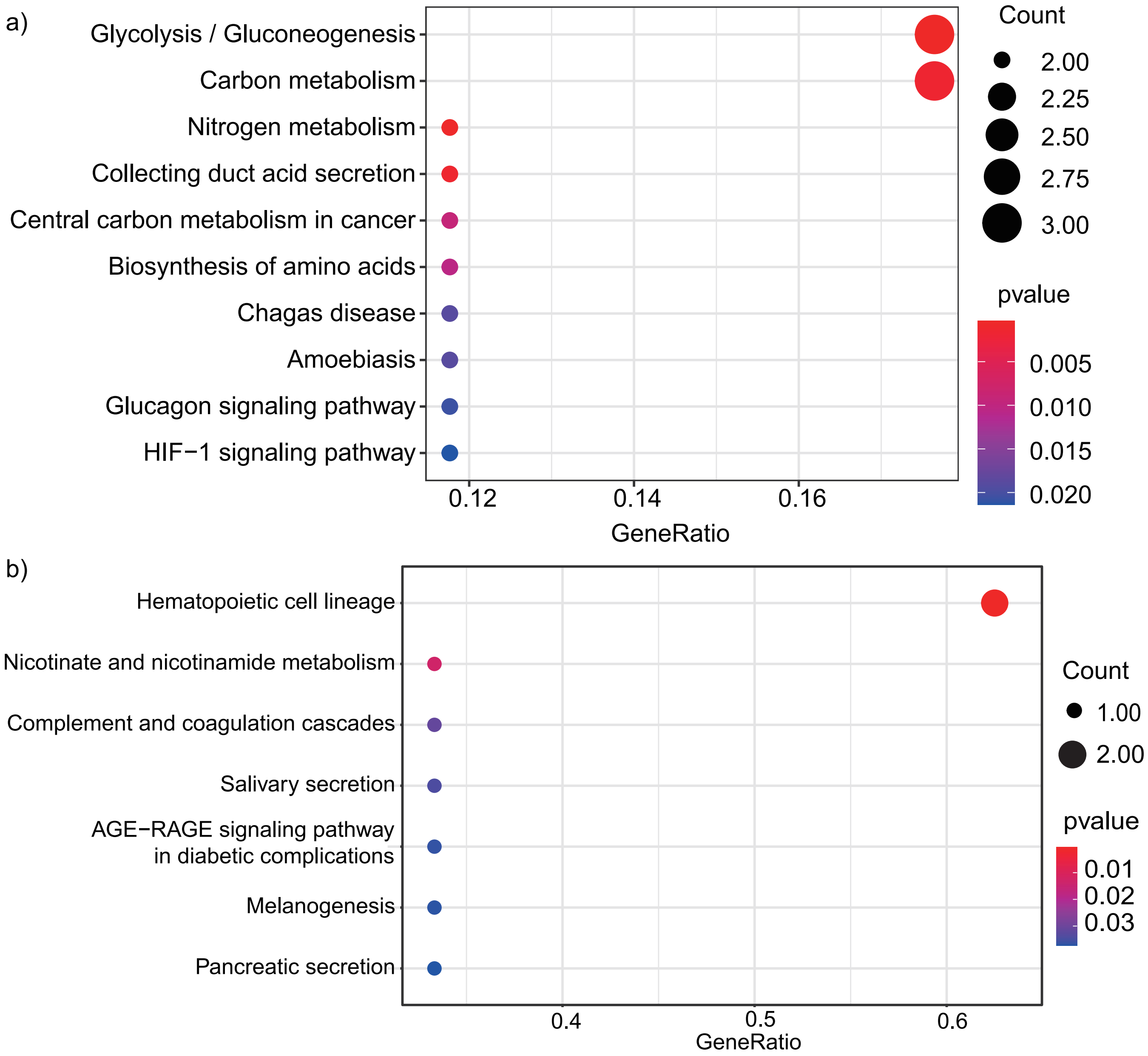

Supplementary Fig. 5

Dot plot of KEGG enrichment of MRM-identified DEPs.
a) KEGG enrichment of MRM-identified DEPs between AMS4k and AMS1k with p-values less than 0.05. Glycolysis/gluconeogenesis, carbon metabolism, and HIF-1 signaling pathways were involved in the development of AMS.
b) KEGG enrichment of the DEPs between the nAMS4k and nAMS1k groups with p-values less than 0.05. The hematopoietic cell lineage was mainly involved in individuals without AMS exposed to high altitude.

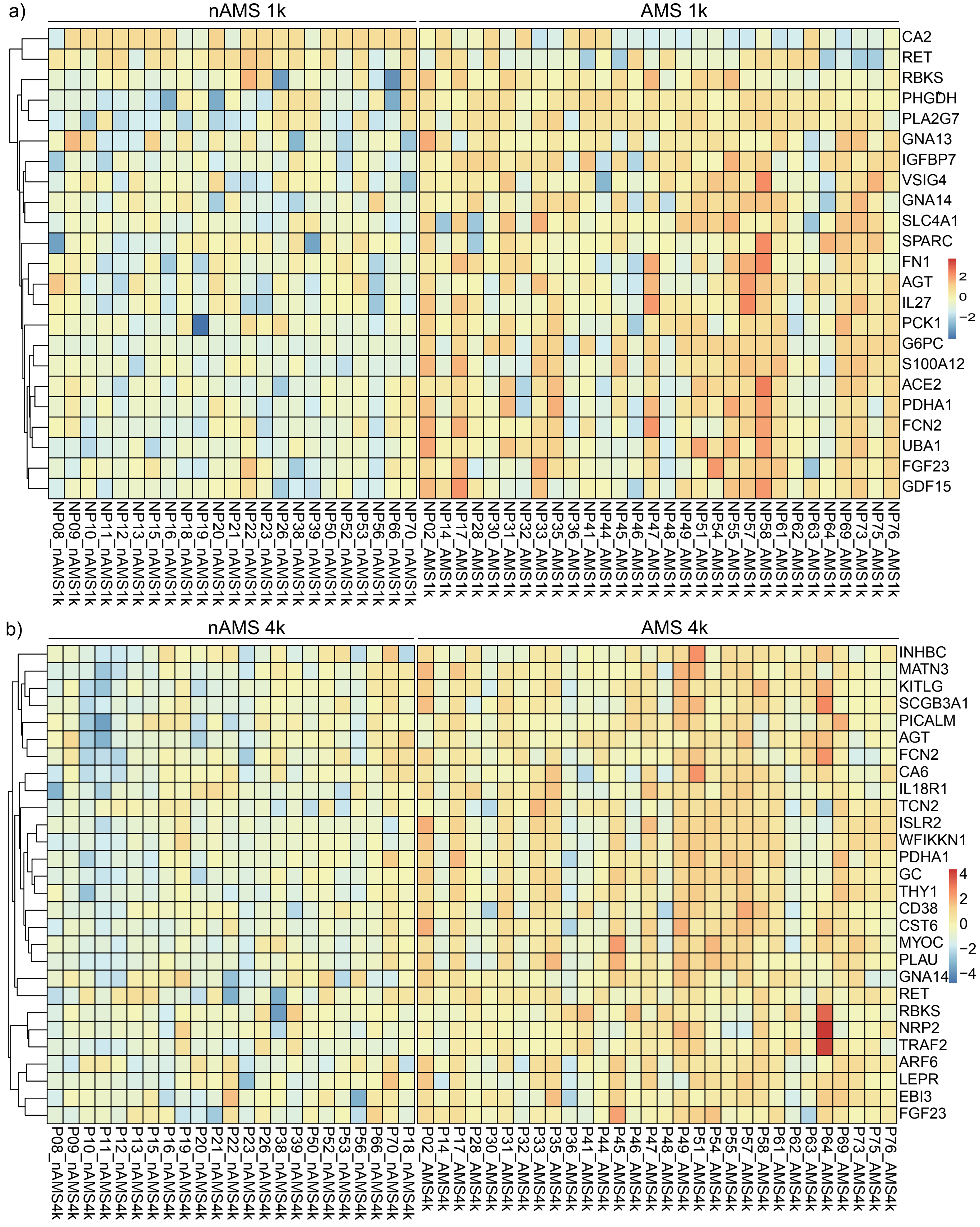

Supplementary Fig. 6

Heatmap of differentially expressed proteins in prediction and diagnosis comparison.
a) Heatmap of 23 differentially expressed proteins (q-values < 0.05) in prediction comparison validated by MRM.
b) Heatmap of 28 differentially expressed proteins (q-values < 0.05) in diagnosis comparison validated by MRM.

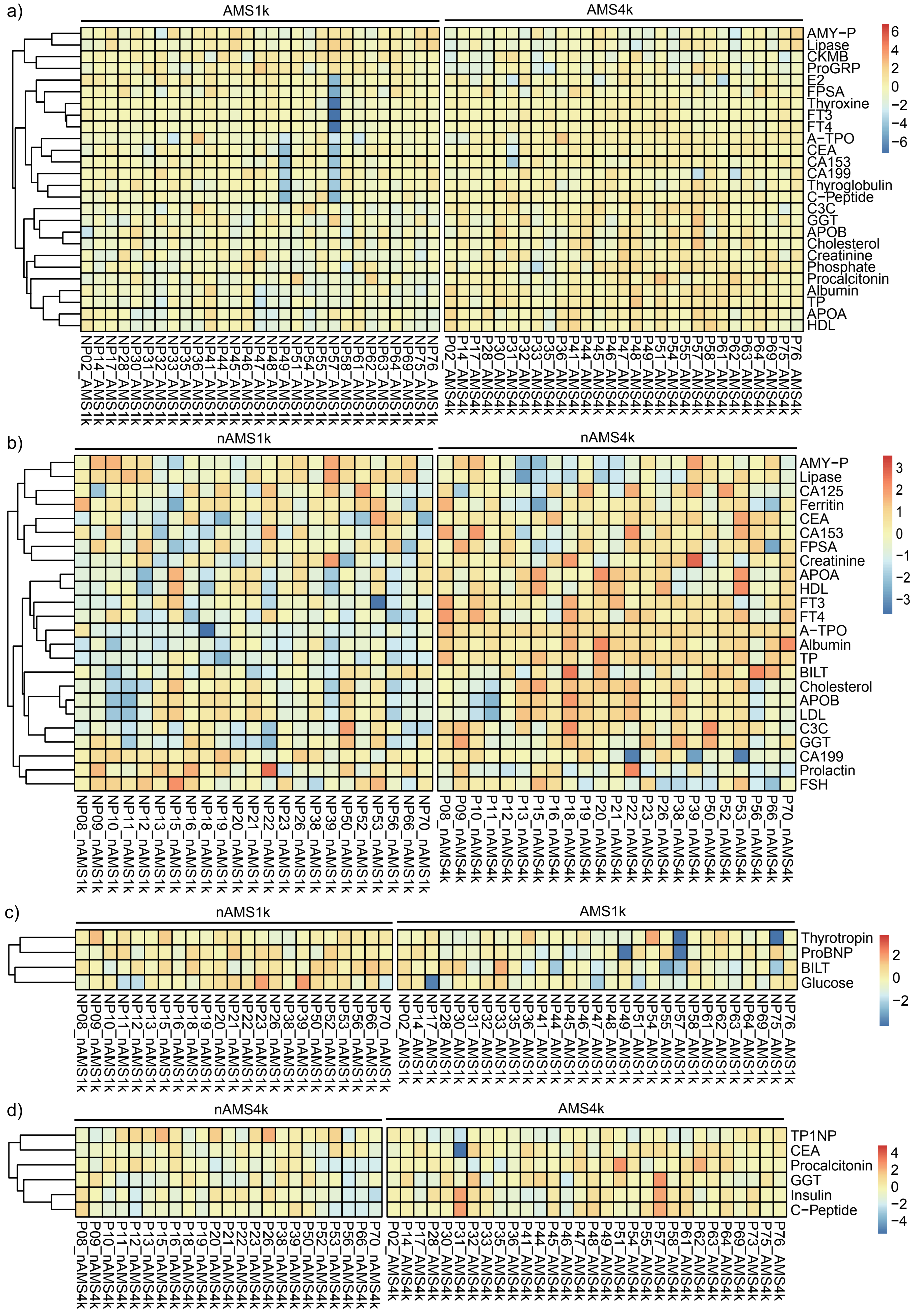

Supplementary Fig. 7

Heatmap of differentially expressed clinical indexes in four comparisons.

a) Heatmap of 26 differentially expressed clinical indexes (q-values < 0.05) in pathogenesis comparison.

b) Heatmap of 24 differentially expressed clinical indexes (q-values < 0.05) in protection comparison.

c) Heatmap of 4 differentially expressed clinical indexes (p-values < 0.05) in prediction comparison.

d) Heatmap of 6 differentially expressed clinical indexes (p-values < 0.05) in diagnosis comparison.

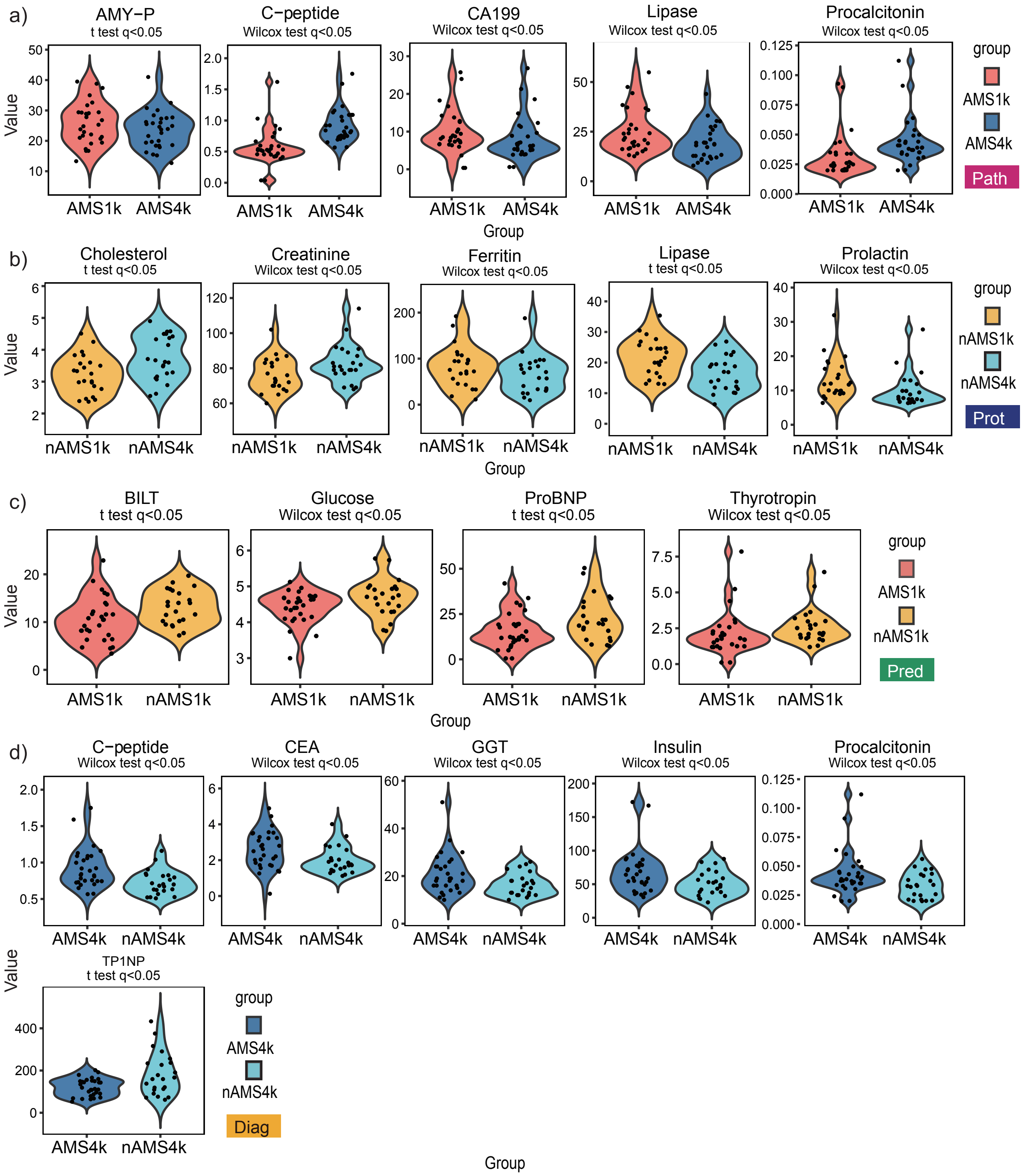

Supplementary Fig. 8

Violin plots of key clinical indexes with statistical significance.
a) Violin plots of five selected clinical indexes showing differences between AMS1k (red violin) and AMS4k (blue violin) (pathogenesis, pink box).
b) Violin plots of five selected clinical indexes showing differences between nAMS4k (light green violin) and nAMS1k (light yellow violin) (protection, dark blue box).
c) Violin plots of clinical indexes showing differences between AMS1k (red violin) and nAMS1k (light yellow violin) (prediction, dark green box).
d) Violin plots of six clinical indexes showing differences between the AMS4k (blue violin) and nAMS4k (light green violin) groups (diagnosis, yellow box).
The comparisons between two groups were assessed by paired or unpaired t-tests and Wilcoxon tests (top of the violin plot) when appropriate.

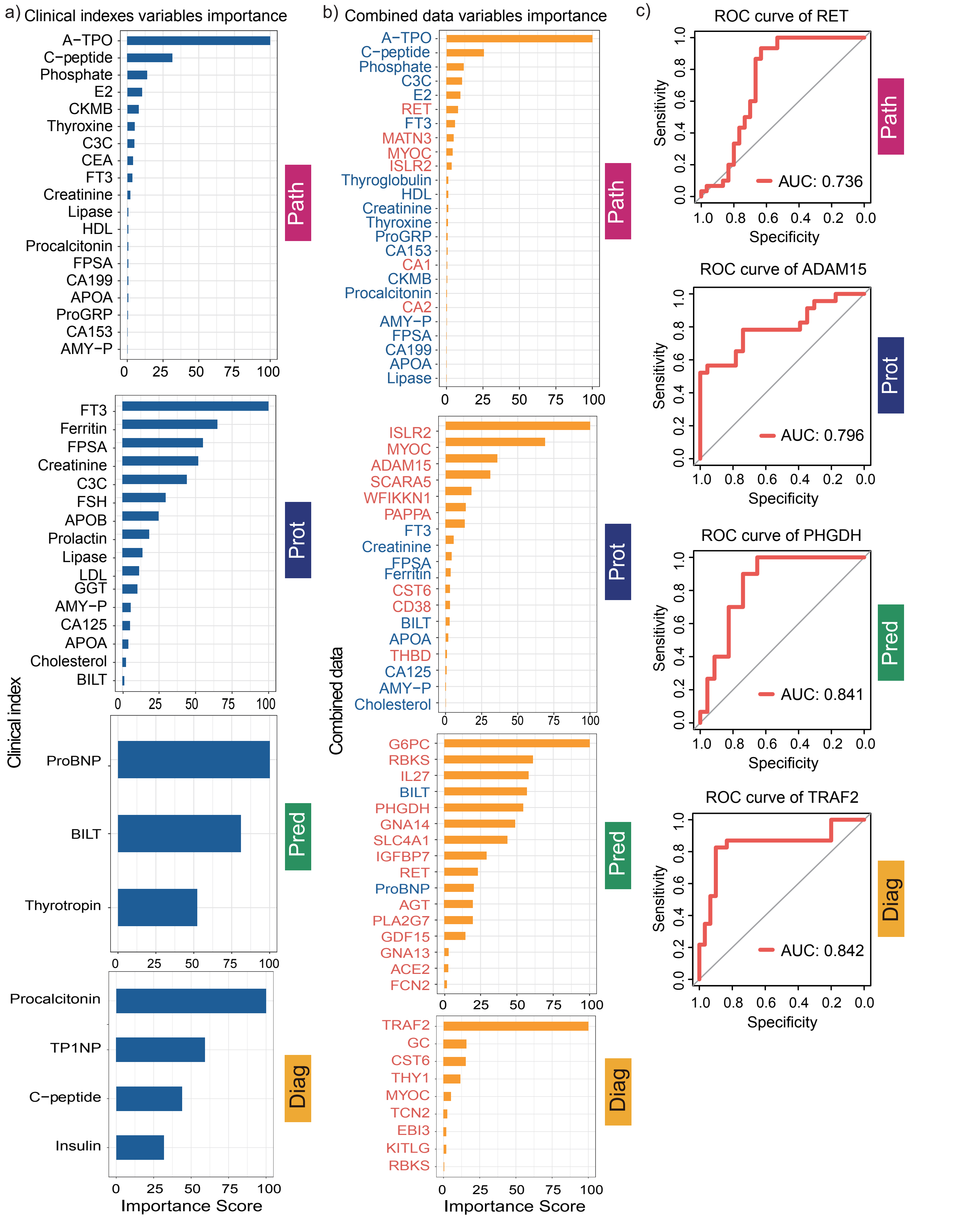

Supplementary Fig. 9

Bar plots of important features in the machine learning models.

a) Bar plots of the importance of the clinical indexes (blue bars) in the pathogenesis (pink box), protection (dark blue box), prediction (dark green box), and diagnosis (yellow box) models.

b) Bar plots of the importance of the combined data (yellow bars) in the pathogenesis (pink box), protection (dark blue box), prediction (dark green box), and diagnosis (yellow box) models. Proteins (orange fonts) accounted for a relatively higher proportion of the important features than clinical indexes (blue fonts) in the four comparison models established using the combined data.

c)ROC curves of RET, ADAM15, PHGDH, and TRAF2 in pathogenesis (pink box), protection (dark blue box), prediction (dark green box), and diagnosis (yellow box) comparison, respectively.

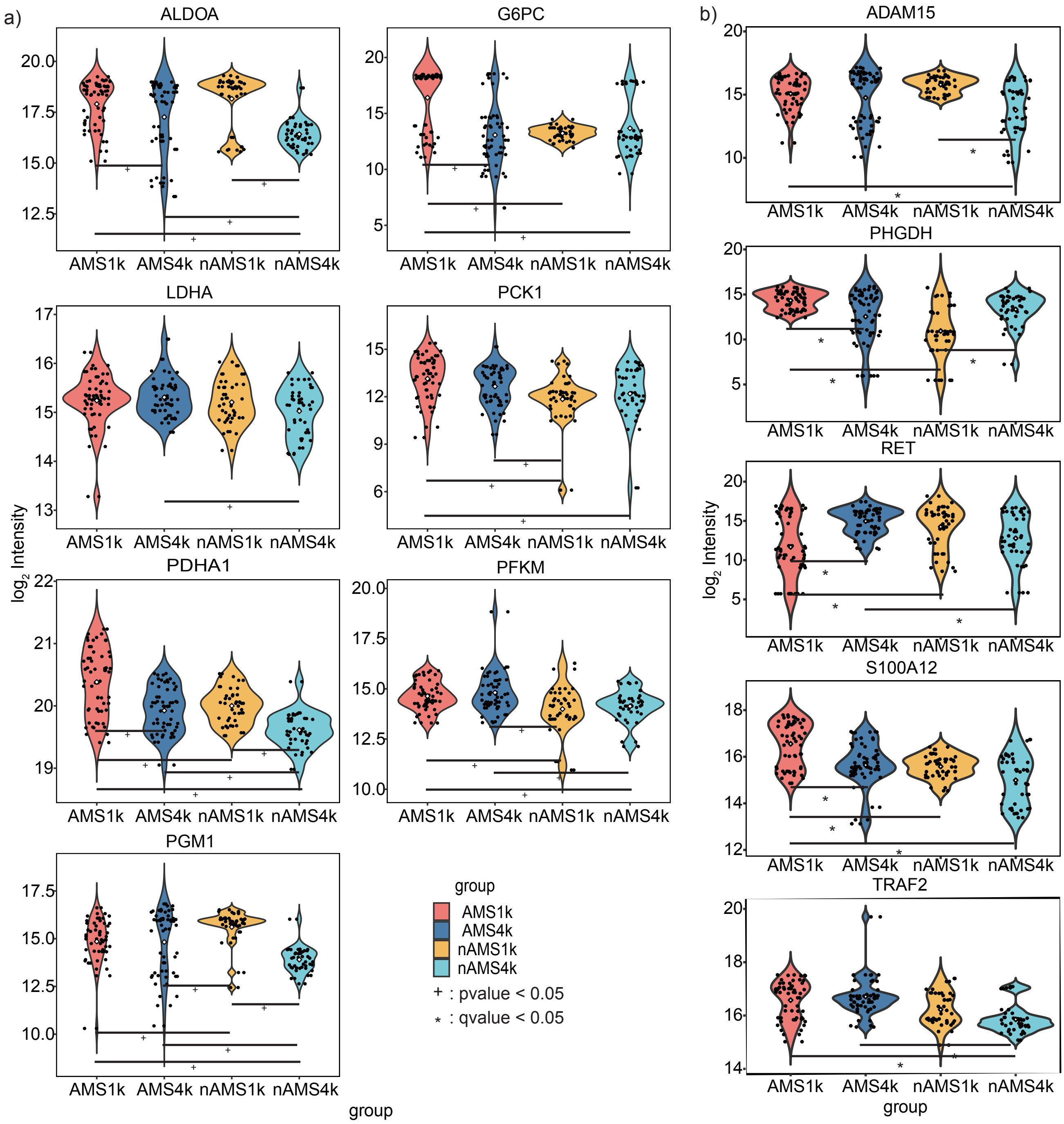

Supplementary Fig. 10

Violin plots of the comparison analysis of key proteins involved in carbohydrate metabolism.
a) Violin plot of 7 proteins related to carbohydrate metabolism. The proteins are presented in the following order: AMS1k (red violin), AMS4k (blue violin), nAMS1k (light yellow violin) and nAMS4k (light green violin).
b) Violin plot of ADAM15, S100A12, PHGDH, RET and TRAF2 proteins in the AMS1k (red violin) and AMS4k (blue violin), nAMS1k (light yellow violin) and nAMS4k (light green violin) groups. The proteins are labeled with * (q-value < 0.05) and + (p-value < 0.05). Each protein related to carbohydrate metabolism was significantly changed in at least one comparison.

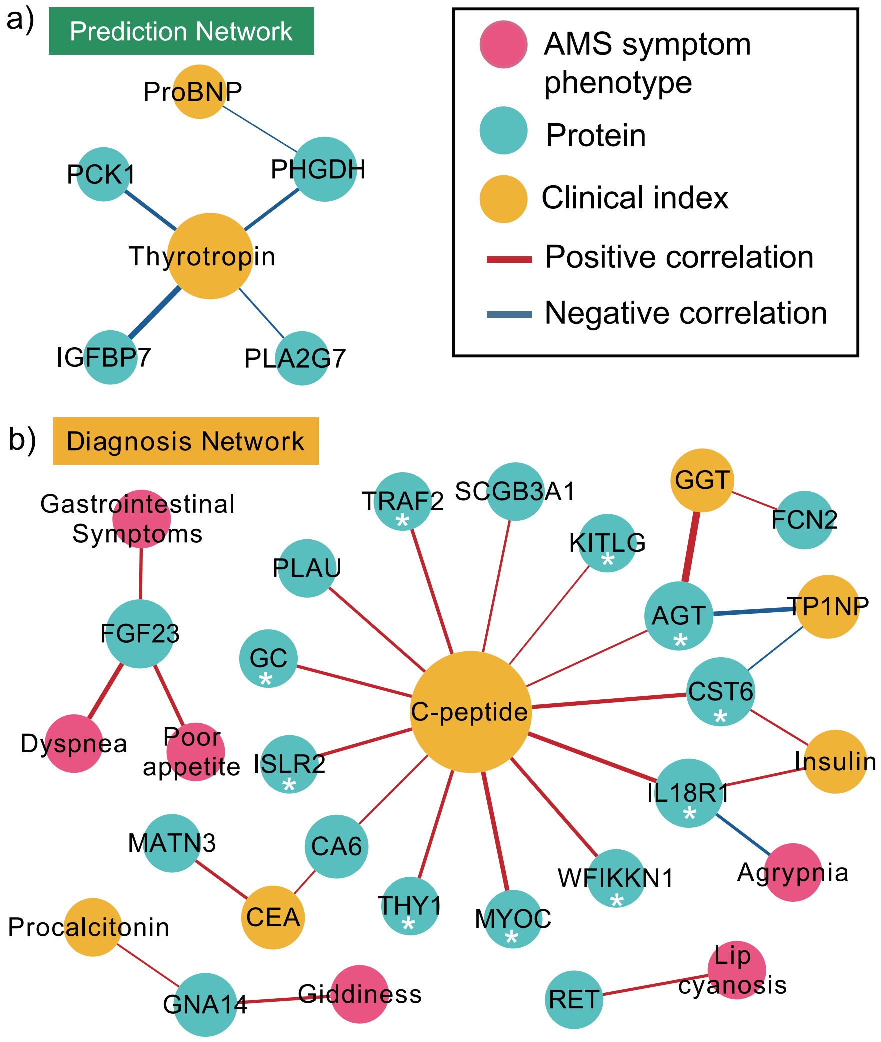

Supplementary Fig. 11

Network visualization of MRM-identified DEPs, clinical indexes and AMS symptom phenotypes in the prediction and diagnosis comparisons.
a) The network of AMS symptom phenotypes (pink dots), DEPs (blue dots), and clinical indexes (yellow dots) were connected based on the Spearman correlation coefficients and the statistical significance between AMS1k and nAMSk (prediction, dark green box).
b) The network of AMS symptom phenotypes (pink dots), DEPs (blue dots), and clinical indexes (yellow dots) was connected based on the Spearman correlation coefficients and the statistical significance between AMS4k and nAMS4k (diagnosis, yellow box). The edges showing a positive correlation (red line) and a negative correlation (blue line) with q-values less than 0.05 are shown. The thickness of the edges corresponds to the absolute value of the correlation coefficients. The size of the node is proportional to the number of edges from the node. C-peptide showed a positive correlation with proteins that were selected by the XGBoost model or differential analysis and labeled with *.
