# Supplementary material for: Proteomic and clinical biomarkers for acute mountain sickness in a longitudinal cohort
Source: Commun Biol. 2022 Jun 6;5:548. doi: 10.1038/s42003-022-03514-6 (PMC9170681; doi:10.1038/s42003-022-03514-6)
Supplement: Supplementary file 3 — Description of Additional Supplementary Files [file 42003_2022_3514_MOESM3_ESM.pdf]

Description of Additional Supplementary Files

File name: Supplementary Data 1
Description: List of AMS symptom phenotypes and clinical indexes. AMS symptom phenotype sheet: Twenty AMSrelated symptom phenotypes were assessed. The gastrointestinal symptoms consist of poor appetite, vomiting, and nausea. LLS was evaluated based on headache, gastrointestinal symptoms, dizziness, and fatigue. Clinical indexes sheet: Overall, 65 clinical indexes were detected.

File name: Supplementary Data 2
Description: List of 1,069 proteins in 12 Olink panels and MRM-validated proteins. PEAmeasured proteins: A total of 1,069 proteins in 12 Olink panels and the detectable rate of each panel are shown. MRM-validated proteins: A total of 102 proteins validated by MRM are shown, including 47 PEA-identified DEPs, and 55 other pathways-related or interest proteins. Peptide fragments: A total of 538 fragments of 102 MRM-validated proteins were included. Transitions: transition lists of 102 MRM-validated proteins.

File name: Supplementary Data 3
Description: Statistical results for differentially expressed proteins verified by PEA and MRM. PEA DEP sheet: The statistical results, such as the statistical method, log2FC and q-value, of DEPs identified by PEA are summarized. The upregulated (red fonts, n = 40) and downregulated (blue fonts, n = 7) proteins identified at the discovery stage are marked. Pathogenesis sheet: The statistical results of proteins identified by MRM with the same regulation trends as those obtained for PEA-identified DEPs between AMS4k and AMS1k are listed (n = 23). Among these proteins, proteins with q-values less than 0.05 are labeled (red fonts, n = 4). Protection sheet: The statistical results of DEPs (n = 2 9, q_value < 0.05) identified by PEA between nAMS4k and nAMS1k are summarized, and proteins showing opposite regulation trends between the AMS4k and AMS1k groups based on PEA-identified DEPs and the proteins validated by MRM are marked (red fonts, n = 5). Prediction sheet: The statistical results of DEPs (n = 23, q_value < 0.05) identified by PEA between AMS1k and nAMS1k are are summarized. The proteins with absolute values of log2FC larger than 0.5 and the same regulation trends as those obtained for the proteins in the comparison between the AMS4k and AMS1k groups are marked (red fonts, n = 6). Diagnosis sheet: The statistical results of DEPs identified by PEA are summarized. Among the DEPs between AMS4k and nAMS4k (n = 28, q_value < 0.05), proteins with absolute values of log2FC larger than 0.5 are marked (red fonts, n = 10).

File name: Supplementary Data 4
Description: GOBP and KEGG enrichment results. GOBP and KEGG enrichment results of the two clusters of proteins identified by PEA between the AMS4k and AMS1k groups and the KEGG enrichment results of the proteins validated by MRM between the AMS4k and AMS1k groups and the nAMS4k and nAMS1k groups.

File name: Supplementary Data 5
Description: Statistical analysis results of 65 clinical indexes. Pathogenesis sheet: Statistical analysis results of clinical indexes between AMS4k and AMS1k. Overall, 18 indexes (black fonts) are also differential indexes (q-values < 0.05) between the nAMS4k and nAMS1k groups. Eight indexes (red fonts) are not differential indexes between the nAMS4k and nAMS1k groups. Protection sheet: Statistical analysis results of clinical indexes between nAMS4k and nAMS1k. Overall, 18 clinical indexes (black fonts) are also differential (q-values < 0.05) between AMS4k and AMS1k groups. Six

clinical indexes (red fonts) are only differential (q-values < 0.05) between Page 14 of 26 nAMS4k and nAMS1k groups, but not between AMS4k and AMS1k groups. Prediction sheet: Statistical analysis results between AMS1k and nAMS1k. Four indexes are differential indexes (p-values < 0.05). Diagnosis sheet: Statistical analysis results between AMS4k and nAMS4k. Six indexes are differential indexes (p-values < 0.05).

File name: Supplementary Data 6
Description: Spearman correlations among 22 AMS symptom phenotypes, differential clinical indexes, and DEPs validated by MRM. The features, Spearman coefficients and p-values are listed in the pathogenesis, prediction, and diagnosis sheets, respectively. The correlations in the pathogenesis comparison with a p-value less than 0.05 are labeled (red fonts, n = 57). The correlations in the prediction and diagnosis comparisons with q-values less than 0.05 are shown.
