# Supplementary material for: Proteomic and clinical biomarkers for acute mountain sickness in a longitudinal cohort
Source: Commun Biol. 2022 Jun 6;5:548. doi: 10.1038/s42003-022-03514-6 (PMC9170681; doi:10.1038/s42003-022-03514-6)
Supplement: Supplementary file 10 — Reporting Summary [file 42003_2022_3514_MOESM10_ESM.pdf]

nature research

Corresponding author(s): Kunlun He, Zhilong Jia

Last updated by author(s): Apr 6, 2022

Reporting Summary

Nature Research wishes to improve the reproducibility of the work that we publish. This form provides structure for consistency and transparency in reporting. For further information on Nature Research policies, see our Editorial Policies and the Editorial Policy Checklist.

Statistics

For all statistical analyses, confirm that the following items are present in the figure legend, table legend, main text, or Methods section.

n/a	Confirmed	
☐	☒	The exact sample size (n) for each experimental group/condition, given as a discrete number and unit of measurement
☐	☒	A statement on whether measurements were taken from distinct samples or whether the same sample was measured repeatedly
☐	☒	The statistical test(s) used AND whether they are one- or two-sided<br>*Only common tests should be described solely by name; describe more complex techniques in the Methods section.*
☒	☐	A description of all covariates tested
☐	☒	A description of any assumptions or corrections, such as tests of normality and adjustment for multiple comparisons
☐	☒	A full description of the statistical parameters including central tendency (e.g. means) or other basic estimates (e.g. regression coefficient) AND variation (e.g. standard deviation) or associated estimates of uncertainty (e.g. confidence intervals)
☐	☒	For null hypothesis testing, the test statistic (e.g. F, t, r) with confidence intervals, effect sizes, degrees of freedom and P value noted<br>*Give P values as exact values whenever suitable.*
☒	☐	For Bayesian analysis, information on the choice of priors and Markov chain Monte Carlo settings
☒	☐	For hierarchical and complex designs, identification of the appropriate level for tests and full reporting of outcomes
☐	☒	Estimates of effect sizes (e.g. Cohen's d, Pearson's r), indicating how they were calculated

Our web collection on statistics for biologists contains articles on many of the points above.

Software and code

Policy information about availability of computer code

Data collection	N.A.
Data analysis	The source code is freely available at https://github.com/Monica1227/AMS_biomarker and Zenodo (10.5281/ZENODO.6418170).

For manuscripts utilizing custom algorithms or software that are central to the research but not yet described in published literature, software must be made available to editors and reviewers. We strongly encourage code deposition in a community repository (e.g. GitHub). See the Nature Research guidelines for submitting code & software for further information.

Data

Policy information about availability of data

All manuscripts must include a data availability statement. This statement should provide the following information, where applicable:

- Accession codes, unique identifiers, or web links for publicly available datasets
- A list of figures that have associated raw data
- A description of any restrictions on data availability

The raw MRM proteomic data analyzed in this study are available at iProX with the corresponding dataset identifier PXD029063. The quantified MRM data is available at the author's GitHub (https://github.com/Monica1227/AMS_biomarker) and Zenodo (10.5281/ZENODO.6418170).

Field-specific reporting

Please select the one below that is the best fit for your research. If you are not sure, read the appropriate sections before making your selection.

☒ Life sciences ☐ Behavioural & social sciences ☐ Ecological, evolutionary & environmental sciences

For a reference copy of the document with all sections, see nature.com/documents/nr-reporting-summary-flat.pdf

Life sciences study design

All studies must disclose on these points even when the disclosure is negative.

Sample size	We used the paired samples and ensured the adaquate power using statistical power analysis with pwr package in R.
Data exclusions	The exclusion criteria of our study design included having any health problems; having any known liver, lung, or cardiovascular disease; a history of migraine or head injury; smoking; and having been to altitudes > 2500 m or exposed to a hypobaric hypoxic environment within the last three months. The exclusion criteria of data we analyzed included: 1) transitions with missing value frequencies greater than 25% ,2)coefficients of variation more than 0.3, 3) samples with less than 75% of observations were eliminated during the correlation analysis. 4) proteins and clinical indexes observed in less than 75% of the samples were deleted, while symptom phenotypes observed in less than 25% of the samples were deleted with the exception of the symptom phenotypes involved in LLS between the AMS4k and AMS1k groups as well as the AMS4k and nAMS4k groups. 5) Features with a difference in detection rate greater than 50% between the two groups were retained. Additionally, features with the same value in each sample were deleted.
Replication	The code to reproduce the figrues and tables in this manuscript is available. MRM-based proteomic analysis was used to validate the candidate biomarkers discoveried in the discovery stage using proximity extension assay.
Randomization	We randomly selected 10 AMS individuals for PEA test in the discovery stage.
Blinding	N.A.

Reporting for specific materials, systems and methods

We require information from authors about some types of materials, experimental systems and methods used in many studies. Here, indicate whether each material, system or method listed is relevant to your study. If you are not sure if a list item applies to your research, read the appropriate section before selecting a response.

Materials & experimental systems

n/a	Involved in the study	
☒	☐	Antibodies
☒	☐	Eukaryotic cell lines
☒	☐	Palaeontology and archaeology
☒	☐	Animals and other organisms
☐	☒	Human research participants
☒	☐	Clinical data
☒	☐	Dual use research of concern

Methods

n/a	Involved in the study	
☒	☐	ChIP-seq
☒	☐	Flow cytometry
☒	☐	MRI-based neuroimaging

Human research participants

Policy information about studies involving human research participants

Population characteristics	A total of 53 Han Chinese male subjects (aged 18-20 years) were recruited in this study.
Recruitment	The exclusion criteria included having any health problems; having any known liver, lung, or cardiovascular disease; a history of migraine or head injury; smoking; and having been to altitudes > 2500 m or exposed to a hypobaric hypoxic environment within the last three months.
Ethics oversight	Ethical approval was obtained from the Chinese PLA General Hospital ethical committee with the approval identifier S2019-035-01, and all protocols followed the established national and institutional ethical guidelines.

Note that full information on the approval of the study protocol must also be provided in the manuscript.
